# Role of ROS and Nutritional Antioxidants in Human Diseases

**DOI:** 10.3389/fphys.2018.00477

**Published:** 2018-05-17

**Authors:** Zewen Liu, Zhangpin Ren, Jun Zhang, Chia-Chen Chuang, Eswar Kandaswamy, Tingyang Zhou, Li Zuo

**Affiliations:** ^1^Radiologic Sciences and Respiratory Therapy Division, School of Health and Rehabilitation Sciences, The Ohio State University College of Medicine, The Ohio State University Wexner Medical Center, Columbus, OH, United States; ^2^Department of Anesthesiology, Affiliated Ezhou Central Hospital, Wuhan University, Ezhou, China; ^3^Department of Pediatrics, Affiliated Ezhou Central Hospital, Wuhan University, Ezhou, China; ^4^Department of Rehabilitation, Affiliated Ezhou Central Hospital, Wuhan University, Ezhou, China; ^5^Interdisciplinary Biophysics Graduate Program, The Ohio State University, Columbus, OH, United States

**Keywords:** antioxidants, cancer, GI diseases, neurodegenerative diseases, oxidative stress, respiratory diseases, vitamins

## Abstract

The overproduction of reactive oxygen species (ROS) has been implicated in the development of various chronic and degenerative diseases such as cancer, respiratory, neurodegenerative, and digestive diseases. Under physiological conditions, the concentrations of ROS are subtlety regulated by antioxidants, which can be either generated endogenously or externally supplemented. A combination of antioxidant-deficiency and malnutrition may render individuals more vulnerable to oxidative stress, thereby increasing the risk of cancer occurrence. In addition, antioxidant defense can be overwhelmed during sustained inflammation such as in chronic obstructive pulmonary diseases, inflammatory bowel disease, and neurodegenerative disorders, cardiovascular diseases, and aging. Certain antioxidant vitamins, such as vitamin D, are essential in regulating biochemical pathways that lead to the proper functioning of the organs. Antioxidant supplementation has been shown to attenuate endogenous antioxidant depletion thus alleviating associated oxidative damage in some clinical research. However, some results indicate that antioxidants exert no favorable effects on disease control. Thus, more studies are warranted to investigate the complicated interactions between ROS and different types of antioxidants for restoration of the redox balance under pathologic conditions. This review highlights the potential roles of ROS and nutritional antioxidants in the pathogenesis of several redox imbalance-related diseases and the attenuation of oxidative stress-induced damages.

## Introduction

Malnutrition is a poor prognostic sign in various diseases, and it is considered a major health concern in developing countries (Muller and Krawinkel, 2005). Reactive oxygen species (ROS) are involved in many important cellular activities including gene transcription, signaling transduction, and immune response. Common ROS include hydroxyl radical (•OH), superoxide (O_2_^•–^) and hydrogen peroxide (H_2_O_2_) (Rogers et al., 2014; Zuo et al., 2015b). An overproduction of ROS can result in oxidative damage to biomolecules such as lipids, proteins, and DNA, which has been implicated in the development of aging as well as various ailments including cancer, respiratory, cardiovascular, neurodegenerative, and digestive diseases. It is reported that the deleterious effects of excess ROS, or oxidative stress (OS), eventually lead to cell death [71]. The body has equipped several mechanisms to counteract the detrimental effects of OS. Antioxidants, either endogenously generated or externally supplied, are capable of scavenging ROS and reducing the oxidation of cellular molecules, thus alleviating OS (Gilgun-Sherki et al., 2001). Antioxidants obtained from the diet are essential in supplying endogenous antioxidants for the neutralization of OS. Indeed, malnutrition and certain antioxidant deficiencies have been correlated with diseases such as chronic obstructive pulmonary disease (COPD) and Crohn’s disease (CD) (King et al., 2008; Alzoghaibi, 2013). A disturbed nutritional and redox balance is frequently observed in these patients. Malnutrition-induced antioxidant deficiency may contribute to increased risks of disease occurrence and poor treatment outcomes (Ames and Wakimoto, 2002; Evatt et al., 2008; Schols et al., 2014). Currently, the clinical awareness of nutritional balance in disease occurrence, progression, and outcomes is limited. An update on the literature review that focuses on the relationship between patients’ nutritional status and disease development is needed. In this review, we will outline the roles of ROS in common OS-associated diseases and aging as well as discuss the effects of nutritional antioxidants as treatments or adjuvants.

## Oxidative Stress and Nutritional Status in Respiratory Diseases

**FIGURE 1:**
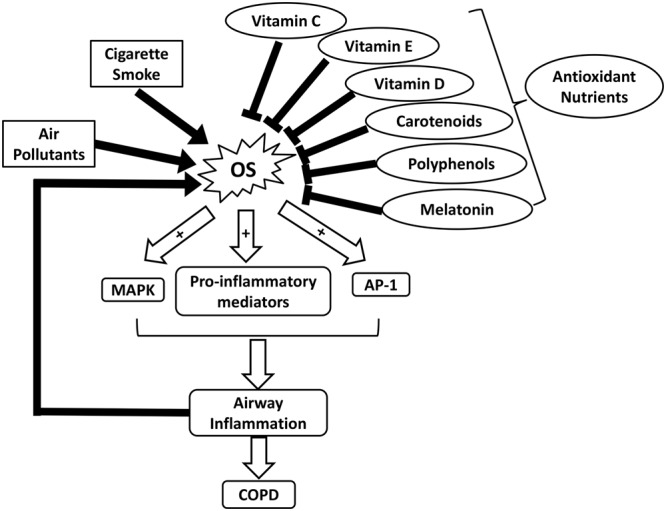
Schematic illustrating the roles of OS and nutrient antioxidants in COPD. AP-1, activator protein-1; COPD, chronic obstructive pulmonary diseases; MAPK, mitogen-activated protein kinase; OS, oxidative stress.

Respiratory diseases such as COPD and asthma have been identified as major health problems due to increased prevalence and mortality worldwide (Masoli et al., 2004; Pauwels and Rabe, 2004). Environmental exposures to air pollutants and cigarette smoke contribute greatly to an increase in OS in COPD (**Figure 1**). The toxicity of oxidants directly damages alveoli and connective tissues of the lungs, exacerbating the development of COPD (van Eeden and Sin, 2013). Excessive ROS formation can also activate inflammatory cells, which in turn generate more ROS in the lungs. This process initiates a vicious cycle of chronic inflammation and OS, as seen in COPD (van Eeden and Sin, 2013). OS is also implicated in the pathophysiology of asthma (Comhair and Erzurum, 2010). Although it remains inconclusive regarding whether increased OS in asthma is a causative factor of the disease or a consequence of inflammation, OS is suggested to play a pivotal role in asthma progression (Cho and Moon, 2010). In bronchial asthma, OS aggravates airway inflammation by activating transcription factors such as nuclear factor-kappa B (NF-κB), mitogen-activated protein kinase (MAPK), activator protein-1 (AP-1), as well as pro-inflammatory mediators (**Figure 1**). Moreover, it enhances airway hyper-responsiveness and stimulates mucin secretion, both of which are associated with severe asthma (Fitzpatrick et al., 2009; Cho and Moon, 2010; Zuo et al., 2016). OS-induced damages in the respiratory system and the reduced antioxidant defenses further lead to an increase in endogenous ROS formation (Jiang et al., 2014).

In addition to OS, low body mass index (BMI) and malnutrition are suggested to correlate with the severity of COPD (King et al., 2008). Underweight COPD patients tend to experience more pulmonary damage, exercise intolerance, and increased mortality rates, in comparison to individuals with normal weights (King et al., 2008; Ferreira et al., 2012). Malnutrition can lead to respiratory muscle mass reduction, which lowers the strength and endurance of these muscles (Ferreira et al., 2012). In addition, decreased intake or availability of dietary antioxidants such as vitamins C and E, carotenoids, and polyphenols, can weaken the antioxidant system and exacerbate disease progression (**Figure 1** and **Table 1**) (Sies et al., 2005; King et al., 2008). A dietary pattern that is rich in vegetables, fruits, fish, and whole grains has been associated with improved pulmonary function and a lower risk of COPD (Varraso et al., 2015). It is suggested that nutritional supplementation enhances respiratory muscle function in malnourished COPD patients, thereby improving their quality of life (Ferreira et al., 1998). For example, Hornikx et al. (2012) reported that high doses of vitamin D supplementation strengthen respiratory muscle function and exercise capacity in individuals with COPD. As the most well-known nutritional antioxidant, vitamin C is capable of reducing oxidative damages and inflammation in the pulmonary system by scavenging excess ROS and activating NF-κB pathway, respectively (Tecklenburg et al., 2007). Furthermore, melatonin, a powerful antioxidant and a regulator of the sleep-wake cycle, can also attenuate OS-related lung deterioration (**Figure 1** and **Table 1**) (Gumral et al., 2009). These findings support the potential use of nutritional antioxidants as an adjuvant to COPD treatment. Similarly, several observational studies suggest that nutritional antioxidants from diets or supplements can improve asthma control and lung function in asthmatic patients (Moreno-Macias and Romieu, 2014). A systematic review has proposed that there is an inverse association between dietary intake of vitamins A and C and incidence of asthma (**Table 1**) (Allen et al., 2009). Vitamin C functions in conjunction with vitamin E to stimulate the regeneration of membrane-bound α-tocopherol from its oxidized states (Moreno-Macias and Romieu, 2014). In addition, dietary carotenoids have been shown to correlate with improved asthma outcomes and lung function (Wood et al., 2012).

**Table 1:** Roles of nutritional antioxidants in human diseases and aging.

Nutritional antioxidant	Common dietary sources	Supplemental effects on human diseases or aging
Anthocyanin	Strawberries, black rice (Peng et al., 2014; Winter et al., 2017).	• Alleviated astrogliosis and preserved neuromuscular junctions and muscle function in ALS (Winter et al., 2017).
		• Extended lifespan in animal models (Peng et al., 2014).
Lipoic acid	Muscle meats, kidney, liver, and heart; low content in fruits and vegetables (Shay et al., 2009).	• Protected neurons against OS-induced mitochondrial dysfunction (Moreira et al., 2010; Zuo and Motherwell, 2013).
Lycopene	Tomatoes, watermelon, papaya, apricot, and pink grapefruit (Sesso et al., 2005; Wood et al., 2012).	• Improved clinical asthma outcomes by suppressing airway inflammation (Wood et al., 2012).
		• Reduced LDL oxidation in blood (Ignarro et al., 2007).
		• Intake of lycopene was inversely correlated with CVD incidence (Kohlmeier and Hastings, 1995; Arab and Steck, 2000; Rao and Agarwal, 2000).
Melatonin	White mustard (seed), black mustard (seed), almond (seed), celery, walnuts, sweet corn, rice (Bonnefont-Rousselot and Collin, 2010).	• Attenuated OS-related lung deterioration in lung diseases (Gumral et al., 2009).
Phytochemicals	Fruits (Mazo et al., 2017)	• Potentially prevent or delay the development of PD (Mazo et al., 2017).
Polyphenols	Fruit, vegetables, coffee, tea, and cereals (Ignarro et al., 2007).	• Higher polyphenol intake was linked with reduced risk of CVD (Vita, 2005).
		• Anti-cancer activity against lung, breast, tongue, gastric, larynx, colon, and prostate cancers (Manikandan et al., 2012; Sak, 2014).
		• Extended lifespan in animal models (Peng et al., 2014).
Resveratrol	Purple wine and peanuts (Anekonda, 2006).	• Protected neurons from Aβ and OS-induced toxicity (Anekonda, 2006; Bellaver et al., 2014).
Selenium	Tuna, oyster, salmon, eggs, green peas, pepper, onion, pork, beef (Navarro-Alarcon and Cabrera-Vique, 2008).	• A combination of selenium and vitamin E protected against oxidative damage in the colon of rats with ulcerative colitis (Bitiren et al., 2010).
Theaflavins	Black tea (Peng et al., 2014).	• Extended lifespan in animal models (Peng et al., 2014).
Vitamin A	Eggs, dairy products, orange-colored fruits, green leafy and yellow-colored vegetables (Tang, 2010).	• Intake of vitamins A and C was inversely associated with the incidence of asthma (Allen et al., 2009).
Vitamin C	Strawberry, Grapefruit, broccoli, and orange (Proteggente et al., 2002).	• Reduced airway inflammation and exercise-induced bronchoconstriction in asthma (Tecklenburg et al., 2007).
		• Intake of vitamins A and C was inversely associated with the incidence of asthma (Allen et al., 2009).
Vitamin D	Fatty ocean fish, sunlight (Holick et al., 2011).	• Improved respiratory muscle function and exercise capability in COPD (Hornikx et al., 2012).
		• Increased the bone mineral density and reduced the risk of hip and other fractures in the elderly (Lips, 2001).
Vitamin E	Wheat germ oil, sunflower oil, hazelnut, and almonds (Reboul et al., 2006)	• Reduced the incidence of CVD death and non-fatal myocardial infarction (Stephens et al., 1996).
		• Attenuated functional decline associated with AD (Sano et al., 1997).
		• A combination of vitamin E and coenzyme Q10 improved energy generation in some cases of Friedreich ataxia (Lodi et al., 2001).
		• A combination of selenium and vitamin E protected against oxidative damage in the colon of rats with ulcerative colitis (Bitiren et al., 2010).

*Aβ, amyloid-β; AD, Alzheimer disease; ALS, amyotrophic lateral sclerosis; COPD, chronic obstructive pulmonary disease; CVD, cardiovascular diseases; LDL, low-density lipoprotein; OS, oxidative stress; PD, Parkinson’s disease.*

The linkage between OS and the development of respiratory diseases suggests a pivotal role of nutritional antioxidants (Romieu, 2005). Vulnerable populations include those with deficiency in dietary antioxidants, increased exposure to environmental sources of oxidants, and poor access to nutritional antioxidants (Moreno-Macias and Romieu, 2014). It is important to note that although antioxidants may help to mitigate the progression of respiratory diseases, antioxidant supplements can act as pro-oxidants or OS inducers if consumed at levels that significantly surpass the recommended dietary intake (Pham-Huy et al., 2008). The potential benefits and risks of nutritional antioxidant supplementation trials in respiratory diseases should be considered on a case-by-case basis. Furthermore, it remains unknown whether OS is a consequence or the causative factor for some pulmonary diseases. Therefore, antioxidant treatment may not be an effective approach to modify disease progression although it may be able to alleviate OS-related symptoms (Margaritelis, 2016).

## Oxidative Stress and Nutritional Status in Cardiovascular Diseases

Cardiovascular diseases (CVD) are the leading cause of mortality in the United States, resulting in nearly one million deaths each year (Madamanchi et al., 2005; Heidenreich et al., 2011). The majority of CVD is correlated with atherosclerosis development, in which OS play a causal role (Madamanchi et al., 2005). Excessive ROS can be generated in vascular cells from NAD(P)H oxidase (Nox), nitric oxide synthases (NOS) uncoupling, and mitochondria, which cause oxidative modifications of low density lipoprotein (LDL) (Azumi et al., 2002; Ambrose and Barua, 2004; Madamanchi et al., 2005). The oxidized LDL (ox-LDL) transported through the arterial lumens induces apoptosis of endothelial cells and smooth muscle cells (SMCs). By taking up ox-LDL, macrophages may transform into foam cells, which secrete growth mediators to attract SMCs into the intima. SMCs can secret extracellular matrix that forms a thin fibrous cap surrounding the fatty streak (Madamanchi et al., 2005; Cachofeiro et al., 2008). With the continuous propagation of SMCs, monocytes, and macrophages, fatty streaks are ultimately converted into more advanced fibrous plaque (Madamanchi et al., 2005), potentially leading to vessel occlusion (Cachofeiro et al., 2008). Further, OS has also been implicated in the development cardiac hypertrophy, ischemic-reperfusion injury, and myocyte apoptosis, all of which may contribute to heart failure (Madamanchi et al., 2005; Zhou et al., 2018).

Considering the implications of ROS in CVD development, numerous studies have been performed to evaluate the effects of nutritional antioxidants in CVD patients. Consumption of fruit and vegetable is found to increase the levels of antioxidants such as carotene and vitamin C in the blood as well as decrease the cholesterol oxidation (Zino et al., 1997; Asplund, 2002). Therefore, the potential benefits of fruits and vegetables in CVD have been broadly investigated. In a meta-analysis consisting of 16 prospective cohort studies and 833,234 participants, CVD-related mortality was found to be inversely correlated with fruit and vegetable consumption (Wang et al., 2014a). Another study involving 2002 patients with coronary atherosclerosis showed that supplementation of natural α-tocopherol (RRR-AT) can significantly reduce the incidence of CVD-related death and non-fatal myocardial infarction (**Table 1**) (Stephens et al., 1996). However, different results are present suggesting no beneficial effect of vitamin supplementation on CVD mortality or morbidity (Kris-Etherton et al., 2004). For example, a meta-analysis study, which involved 81,788 participants, reported that daily supplementation of either vitamin E at a dose of 50–800 IU or β carotene at a dose of 15–80 mg did not decrease the mortality associated with CVD (Vivekananthan et al., 2003). Therefore, vitamin E and β carotene may not be the only active constituent of fruits and vegetables that exert cardiovascular protective effects. Instead, other antioxidant compounds such as lycopene and polyphenols could play a more important role in the protection against CVD as will be discussed below (Ignarro et al., 2007). Furthermore, the inconsistency in treatment outcomes are likely associated with antioxidant formulation. Most of the trials reporting inefficacy of vitamin E have used all-racemic α tocopherol, which is a major constituent of synthetic vitamin E (Hoppe and Krennrich, 2000; Madamanchi et al., 2005). By contrast, RRR-AT, the natural form of vitamin E has been associated with better treatment effects (Hoppe and Krennrich, 2000; Madamanchi et al., 2005). Thus, further research is needed to address the difference between RRR-AT and all-racemic α tocopherol in terms of their therapeutic efficacy. Additionally, the complicated redox mechanisms of antioxidant are far from clear now. Some of the antioxidant such as vitamin C may exhibit prooxidant properties when administrated at high doses (Madamanchi et al., 2005). This could partially explain why some of the trials using antioxidant supplementation failed to show any protective effect.

Lycopene is a natural dietary antioxidant most abundant in tomatoes. An inverse association was found between CVD incidence and consumption of either tomatoes or lycopene (Kohlmeier and Hastings, 1995; Arab and Steck, 2000; Rao and Agarwal, 2000). This could be attributed to the protective effects of lycopene against LDL oxidation by inhibiting cholesterol synthesis and improving LDL degradation (**Table 1**) (Ignarro et al., 2007). An early population-based study was conducted to evaluate the relationship between the risk of myocardial infarction and the status of three types of carotenoids including lycopene, α carotene, and β carotene, respectively. It was found that only lycopene had significant protective effects (Kohlmeier et al., 1997). Therefore, lycopene may be one of the primary contributors that underlie the protective mechanisms of vegetable consumption against CVD (Kohlmeier et al., 1997). Additionally, polyphenols are the most abundant antioxidants in human diet (∼1 g/d), widespread in fruit, vegetables, coffee, tea, and cereals (Ignarro et al., 2007). Epidemiologic studies found a significantly reduced risk for CVD with higher polyphenol intake (**Table 1**) (Vita, 2005). Beverages rich in flavonoid such as tea can markedly improve endothelial function. However, tea consumption did not reduce the oxidative markers in the blood. So it remains elusive whether this beneficial effect of tea is elicited by its antioxidant effects (Vita, 2005). Indeed, increasing evidence has suggested that the protective effects of polyphenols are not solely contributed by their antioxidant ability but more likely correlated with their anti-inflammatory effects as well as the regulation of vasodilation and apoptosis of endothelial cells (Quinones et al., 2013).

## Oxidative Stress and Nutritional Status in Neurodegenerative Disorders

Neurons are particularly vulnerable to OS-induced damage due to their weakened antioxidant defense system, high demand for oxygen consumption, and abundant polyunsaturated fatty acid content in their cell membranes (Rego and Oliveira, 2003). A growing number of studies indicate that ROS may be generated via different mechanisms and play complex roles in the development of neurodegenerative diseases such as Alzheimer’s disease (AD), Huntington’s disease (HD), amyotrophic lateral sclerosis (ALS), Parkinson’s disease (PD), and spinocerebellar ataxia (SCA) (Davila and Torres-Aleman, 2008; Hakonen et al., 2008; Patten et al., 2010; Blesa et al., 2015; Covarrubias-Pinto et al., 2015; Zuo et al., 2015b). AD is a major cause of dementia in elderly (Harman, 2006). Although the exact pathogenesis of AD remains elusive, aging-related progressive increase in OS has been considered a chief contributor to the formation of AD lesions (Harman, 2006; Pimplikar et al., 2010). Evidence has suggested that oxidative events occur prior to the onset of plaque pathology and amyloid-β (Aβ) accumulation, which further supports the critical roles of OS in the initiating stage of AD (Lin and Beal, 2006; Wang et al., 2014b). In AD, OS modulates JNK/p38 MAPK pathways, leading to the accumulation of Aβ and the hyper-phosphorylation of tau proteins (Patten et al., 2010). HD is an autosomal dominant inherited disease, which is caused by a mutated expansion of CAG repeat in exon 1 of *HD* gene and its resulted mutant protein product “huntingtin” (mHtt) (Browne and Beal, 2006). OS is not the initiation factor of HD. However, severe OS is a typical feature of HD and may contribute to the increased DNA oxidation in the HD brain (Browne and Beal, 2006). OS-induced mitochondrial dysfunction is commonly observed in HD and the impairment of respiratory chain can exacerbate ROS formation (Browne and Beal, 2006). Furthermore, mitochondrial aconitase, an important tricarboxylic acid (TCA)-cycle enzyme, is significantly impaired in HD. The decline in aconitase activity is thought to be caused by ROS-induced oxidation of Fe-S cluster within aconitase (Browne and Beal, 2006). As a result, OS is responsible for the metabolic defects seen in HD (Browne and Beal, 2006). In HD, OS is also related to decreased expression of glucose transporter (GLUT)-3, which results in the inhibition of glucose uptake and the over-accumulation of lactate (Covarrubias-Pinto et al., 2015). It remains inconclusive whether OS is an initiator or consequence of neurodegeneration in PD. However, excessive ROS production is a critical component of the mechanisms underlying PD progression (Jenner, 2003). Loss of antioxidant defense, especially glutathione (GSH) content is found early in PD although the cause remains unknown (Jenner, 2003). High levels of oxidation of protein, DNA, and lipids are observed in PD. The toxic products from the oxidative damage may lead to neural cell death (Jenner, 2003). In the substantia nigra pars compacta (SNc) of PD patients, reduced activity of Complex I in the mitochondrial respiratory chain contributes to excessive ROS generation and consequently induces the apoptosis of dopaminergic neurons (Blesa et al., 2015). In ALS, superoxide dismutase (SOD) 1 mutation and mitochondrial degeneration represent one of the major mechanisms underlying ALS pathology (Rotunno and Bosco, 2013). Specifically, significant vacuolar degeneration of mitochondria was observed just before the death of neuron in *SOD1* mutant mice, indicating that mitochondrial dysfunction initiates the onset of ALS (Lin and Beal, 2006). Mutant SOD1 has been shown to abnormally interact with mitochondria, leading to cytochrome *c* release and activation of apoptosis (Lin and Beal, 2006). A decline in antioxidant capability due to SOD1 mutation is potentially associated with motor neuron degeneration (Zuo et al., 2015b). In addition, elevated OS can inhibit neuroprotective IGF-I/AKT pathways, resulting in neuron cell dysfunction (Davila and Torres-Aleman, 2008). Furthermore, marked mitochondrial alterations caused by OS have been suggested to be involved in the development of SCA (Stucki et al., 2016).

Considering the complex roles of OS in neurodegenerative disorders, the regulation of cellular ROS levels may represent a potential treatment to impede neurodegeneration and alleviate associated symptoms (Uttara et al., 2009). Clinical evidence indicates that neurodegenerations can be ameliorated upon proper intake of natural or supplementary antioxidants (Zandi et al., 2004). On the other hand, a lack of major antioxidants due to malnutrition, which is implicated in various neurodegenerative diseases, can worsen the progress of neurological conditions (Brambilla et al., 2008; Tsagalioti et al., 2016). For example, vitamin D deficiency has recently emerged as one of the contributing factors leading to aberrant neurological development. Vitamin D is an essential antioxidant that regulates calcium-mediated neuronal excitotoxicity and the induction of neurotransmitters and synaptic structural proteins (Mpandzou et al., 2016; Wang et al., 2016). Wang et al. (2016) suggested that inadequate vitamin D in serum is highly associated with the loss of dopaminergic neurons in PD brains and increased risk of PD. Neurological impairments have also been manifested in individuals with a vitamin B deficiency. Multiple vitamin B (e.g., B1, B3, and folate) deficiencies are implicated in the pathophysiology of numerous neurodegenerative diseases such as PD and AD (Sechi et al., 2016).

Rutin, resveratrol, and vitamin E, which target ROS-mediated cascades such as JNK and NF-κB, have yielded some positive outcomes in improving neurodegeneration both *in vitro* and *in vivo* (Zuo et al., 2015a). In a rat brain, vitamin E was found to be more effective in modulating OS than vitamins A and C (Zaidi and Banu, 2004). Accordingly, a 2-year administration of vitamin E at a dose of 2000 IU per day has been shown to reduce the functional decline associated with AD (Sano et al., 1997). The combination of vitamin E and coenzyme Q10 improves energy generation in some cases of Friedreich ataxia by attenuating OS and restoring mitochondrial function (Lodi et al., 2001). In addition to vitamins, phytochemicals, another type of bioactive compounds that can be found in fruits and vegetables, exhibit high antioxidant capacity with potential neuroprotective effects against PD (Mazo et al., 2017). Anthocyanin derived from strawberries possesses anti-oxidative, anti-inflammatory, and anti-apoptotic abilities. It has been reported to alleviate astrogliosis and preserve neuromuscular junctions and muscle function, serving as a possible therapeutic agent for ALS and other neurodegenerative diseases (Winter et al., 2017). During AD progression, resveratrol’s potential in protecting neurons from Aβ and OS-induced toxicity shows promising therapeutic applications (Anekonda, 2006; Bellaver et al., 2014). Lipoic acid (LA) is shown to enhance GSH generation and deplete lipid peroxide, thus protecting neurons against OS-induced mitochondrial dysfunction (**Table 1**) (Moreira et al., 2010; Zuo and Motherwell, 2013). Long-term administration of MitoQ, a mitochondria-target antioxidant, also significantly restores mitochondrial functions in Purkinje cells and alleviates SCA1-related symptoms such as motor incoordination (Stucki et al., 2016).

Numerous studies have been performed to investigate the therapeutic effects of natural antioxidants on neurodegenerative disorders; however, mixed results have been yielded (Dias et al., 2013; Yan et al., 2013). For instance, despite the seeming effectiveness of vitamin E, a study has showed that vitamin E intake for 5 months failed to elevate vitamin E levels in ventricular cerebrospinal fluid of PD patients (Pappert et al., 1996). ROS formation is subtly regulated by antioxidant defense systems within the human body (Zuo et al., 2015b). Hence, single antioxidant intake could not be sufficient to resist OS under pathophysiological conditions and could result in cellular damage (Murphy, 2014). In this regard, a combined use of various nutritional antioxidants should be considered. Importantly, the simple dichotomy in redox biology comprised of good antioxidants and bad ROS is regarded as untenable. It is now well accepted that a small amount of ROS is essential to activate redox-sensitive signaling pathways, while excessive ROS can lead to detrimental effects (Margaritelis et al., 2016). The different characteristics and sources of ROS may define their specific roles in regulating cellular activities (Winterbourn and Hampton, 2008; Margaritelis et al., 2016). Numerous studies have stressed the need for a more precise description of the metabolism of ROS in aspects of quantity, reactivity, location, and reaction kinetics (Winterbourn and Hampton, 2008; Forman et al., 2014; Margaritelis et al., 2016). However, most of the exogenously administrated antioxidants are non-selective and distributed uniformly across various parts of the cells or tissues (Margaritelis et al., 2016). The lack of specificity of antioxidants may account for their inefficacy in treating OS-related diseases. It is thus imperative that researchers focus on developing novel and targeted antioxidants such as mitoQ and Nox inhibitors to improve the precise therapeutic effects of antioxidants in future studies (Altenhofer et al., 2015; Margaritelis et al., 2016).

## Oxidative Stress and Nutritional Status in Cancer

ROS are involved in all three stages of cancer development, namely initiation, promotion, and progression (Khandrika et al., 2009; Wells et al., 2009; Katakwar et al., 2016). In the initiation stage, ROS-induced DNA mutations can accumulate if they are not repaired in cancerous tissues (Poulsen et al., 1998). Excessive ROS production may lead to oncogenic mutation of DNA, potentially contributing to the onset of cancer (Valko et al., 2006). In addition, cancer cells are characterized by more ROS production than normal cells due to an altered metabolism and increased energy demand (Sosa et al., 2013). ROS-induced OS in carcinoma cells may promote cancer growth by triggering cell growth signaling, enhancing tumor resistance to therapies, increasing blood supply to tumors, and promoting metastasis (Brown and Bicknell, 2001). ROS promote the expansion of cancerous cells by modifying the genes related to apoptosis, cell proliferation and transcription factors (Trueba et al., 2004). ROS also upregulate antiapoptotic genes and downregulate proapoptotic proteins via PI3K/AKT and ERK/MEK pathways (McCubrey et al., 2007). In the progression stage of cancer development, ROS contribute to the upregulation of matrix metalloproteinases, inhibiting the action of anti-proteases and angiogenesis, eventually leading to metastasis (Maulik, 2002; Mori et al., 2004; Shinohara et al., 2010).

A depletion of endogenous antioxidants or a disruption of redox equilibrium may lead to cancer development. Research has shown that 35% of cancer can be prevented by dietary modifications (Doll and Peto, 1981; Rayman, 2005). Fruits and vegetables, which are rich in antioxidants, exert a protective effect against several different types of cancers (Soerjomataram et al., 2010; Turati et al., 2015). Plant foods that contain polyphenols have proven to be effective antioxidant agents for the body (Barrajon-Catalan et al., 2010; Paredes-Lopez et al., 2010; Sreelatha et al., 2012; Cordero-Herrera et al., 2013). They have been shown to possess anti-cancer activity which is effective against lung, breast, tongue, gastric, larynx, colon, and prostate cancers (**Table 1**) (Manikandan et al., 2012; Sak, 2014). Fruits containing higher phenolic content have stronger antioxidant properties since they can induce hydroxyl group substitution in the aromatic rings of phenol compounds (Sun et al., 2002; Rokayya et al., 2014). Polyphenols induce apoptosis of cancer cells, inhibit proliferation of mutated cells, reduce production of cyclooxygenase-2 (COX-2), and downregulate cancer gene expression (Gloria et al., 2014; Li et al., 2014; Xie et al., 2014; Zhang et al., 2015). Moreover, nutrients such as vitamins and minerals can reduce cancer risk by eliciting antioxidant action, inhibiting proliferation of cancerous cells, maintaining DNA methylation, and promoting cell-cycle arrest (Pathak et al., 2003; Rayman, 2005). In individuals previously treated for cancer, a healthy diet rich in fruits and vegetables can modify biologic markers of cancer progression (Jones and Demark-Wahnefried, 2006). Healthy plant foods have shown to reduce the risk of death after being diagnosed with breast (Vrieling et al., 2013; George et al., 2014), head and neck (Arthur et al., 2013), and rectal cancers (Pelser et al., 2014). A high vegetable diet has been shown to be effective in reducing breast cancer recurrence for patients on tamoxifen (Gold et al., 2009; Thomson et al., 2011).

Vitamins such as Vitamin A and E have a preventive effect against oral cancer (Garewal, 1995). Selected micronutrients (vitamin D, carnitine, and selenium) have been shown to improve compliance and prognosis, patients’ quality of life, and reduced adverse effects of cancer treatments (Block et al., 2008; Grober et al., 2015). However, limited evidence supports the effectiveness of vitamins and minerals in cancer prevention (Fortmann et al., 2013), and such nutritional regimens are not currently recommended for practice in healthy individuals (World Cancer Research Fund/American Institute for Cancer Research, 2007). Additionally, there is a lack of randomized control trials investigating diets and cancer due to difficulty in whole diet interventions as well as ethical issues in the proposed research (Norat et al., 2015). Hence, current recommendations are based on the effectiveness of a healthy diet (rich in fruits, vegetables, and grains, and low on red meat and alcohol) and lifestyle on reducing cancer risk (Norat et al., 2015).

## Oxidative Stress and Nutritional Status in Digestive Diseases

**FIGURE 2:**
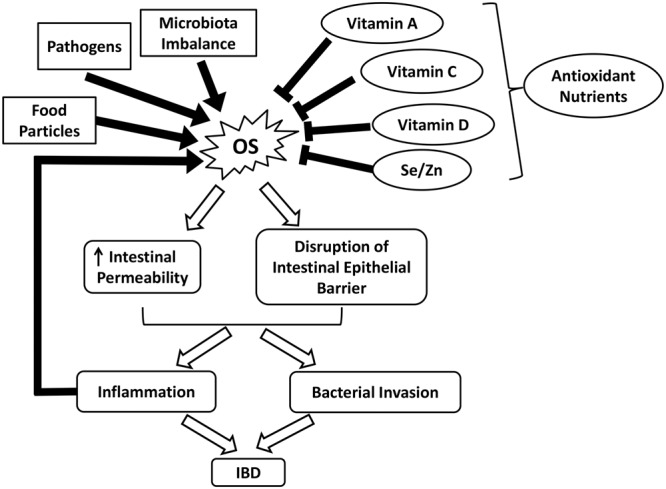
Schematic illustrating the roles of OS and nutrient antioxidants in IBD. IBD, inflammatory bowel diseases; OS, oxidative stress; Se, selenium; Zn, zinc.

It is well established that intestinal inflammation-associated OS plays an essential role in the pathophysiology of various gastrointestinal (GI) diseases, such as inflammatory bowel diseases (IBD) (Balmus et al., 2016). Although the exact etiology of IBD remains unclear, the underlying pathologies can be partially attributed to excess ROS formation (Zhu and Li, 2012; Bhattacharyya et al., 2014). Due to the presence of food particles, pathogens, or microbiota imbalance, the GI tract may become irritated, generating excess ROS and compromising endogenous antioxidant defenses (Moura et al., 2015). OS disrupts the intestinal epithelial barrier and increases intestinal permeability, further exacerbating inflammation (**Figure 2**) (Balmus et al., 2016). IBD, which is comprised of CD and ulcerative colitis (UC), is characterized by chronic and prominent inflammation associated with OS in the GI tract (Balmus et al., 2016). Elevated levels of pro-inflammatory mediators such as platelet activating factor (PAF) and leukotriene B_4_ (LTB_4_) observed in the mucosal samples from active IBD patients have been shown to trigger the release of cytotoxic reactive oxygen metabolites by overstimulating phagocytes (Ingraham et al., 1982; Sharon and Stenson, 1984; Wallace and Chin, 1997). Moreover, myeloperoxidases are released during the massive infiltration of polymorphonuclear neutrophils and macrophages into the inflamed mucosa, producing hypochlorous acid, a potent oxidizing agent, via the metabolism of H_2_O_2_. Other sources of ROS include enzymes such as cyclooxygenase, xanthine oxidase, and 5-lipoxygenase that reside in the intestinal mucosa (Alzoghaibi, 2013).

Despite ROS overproduction, a deficiency in dietary and enzymatic antioxidants also contributes to the development of OS (Alzoghaibi, 2013). For example, low levels of enzymatic antioxidants and vitamins have been observed in patients with CD, which is partly due to malnutrition (Buffinton and Doe, 1995; Alzoghaibi, 2013). In malnourished IBD patients, the reduced dietary intakes of fruits and vegetables greatly influence the concentration of carotenoid (vitamin A) (Balmus et al., 2016). Vitamin C, which helps to repair and protect mucosal lining against detrimental insults, is depleted in peptic ulcers and gastritis (Aditi and Graham, 2012). Notably, the increased incidence of vitamin D deficiency in CD patients is highly associated with skeletal morbidity and a worsened quality of life (**Figure 2**) (van Hogezand and Hamdy, 2006; Alastair et al., 2011). Persistent OS can damage the intestinal barrier and increase the permeability of GI epithelium via lipid peroxidation and tight junction disruption. This alters the composition of commensal microbiota in the GI tract and interrupts their ability to establish colonization resistance, thus promoting the invasion of pathogenic bacteria (Buffie and Pamer, 2013; Moura et al., 2015). Such infections further aggravate ROS production and inflammation and potentially increase the risk of inflammatory bowel syndrome (Zhu and Li, 2012).

Considering a strong indication of ROS elevation in IBD and other GI diseases, the adjuvant or treatment potential of antioxidants are largely investigated. Antioxidant applications have been shown to restore redox balance, thereby attenuating intestinal damages and maintaining GI health (Bhattacharyya et al., 2014). For example, studies have shown that CuZn-SOD and 5-aminosalicylic acid effectively alleviate mucosal injuries in CD by scavenging or inducing rapid decomposition of ROS (Emerit et al., 1989; Couto et al., 2010; Alzoghaibi, 2013). In a randomized placebo-controlled study, 3 months of oral antioxidant supplementation markedly improved the serum antioxidant status in CD patients in remission. The combination of antioxidants with *n*-3 fatty acids further attenuated pro-inflammatory activities, thus serving as a potential treatment for CD (Geerling et al., 2000). Compared to supplements, dietary intakes of antioxidants from natural fruits and vegetables may be a safer approach to avoid overconsumption. Inappropriate antioxidant application can be harmful by scavenging of physiological ROS (Bjelakovic et al., 2004; Poljsak et al., 2013). Foods rich in micronutrients such as α-tocopherol (vitamin E) and minerals have been reported to be beneficial in alleviating ROS damage. For example, selenium and zinc interact with GPx and SOD, respectively, to combat OS. The combination of selenium and vitamin E has demonstrated protective effects against oxidative damage in the colon of UC rats (**Figure 2** and **Table 1**) (Bitiren et al., 2010). Several functional foods may be beneficial for IBD without undesirable effects.

## Oxidative Stress and Nutritional Status in Aging

Free radical theory, which was first proposed by Harman in 1956, suggests that aging is process related with progressive and irreversible accumulation of oxidative damage in the cells (Harman, 1956; Mariani et al., 2005). A shift of redox balance toward a more oxidized status is noted in aging cells, as indicated by decreased GSH/GSSG ratio. This alteration of redox profile may blunt cellular capability of buffering ROS produced both under physiological conditions and in response to external stress (Kregel and Zhang, 2007). Excessive ROS accumulation can directly damage DNA, protein, and lipids, which disturbs normal cellular function (Zuo et al., 2015b). Mitochondrial DNA (mtDNA) is particularly susceptible to OS and the mutation of mtDNA has been closely linked with the aging process (Trifunovic et al., 2004). It was reported that mice with somatic mtDNA mutation exhibited an earlier onset of aging-related features such as hair loss, osteoporosis, and decreased subcutaneous fat as well as a shorter lifespan (Trifunovic et al., 2004). Exposure to high levels of ROS can also accelerate telomere shortening, which ultimately triggers cellular senescence (Kregel and Zhang, 2007). For example, fibroblast cells cultured under high OS showed increased rate of telomere shortening and a reduced lifespan (Vonzglinicki et al., 1995). Additionally, aging-associated OS could be responsible for the chronic systematic inflammation as commonly seen in the elderly via the activation of NF-κB (Chung et al., 2009). NF-κB is a key regulator for inflammatory factors such as tumor necrosis factor-alpha (TNF-α), interleukin (IL)-1β, and IL-6 (Chung et al., 2009). OS-induced NF-κB signaling is short-lived under normal conditions in contrast to chronic activation during aging (Chung et al., 2009). The persistent low-level inflammation could be responsible for the development of age-related diseases such as atherosclerosis, cancer, and dementia (Chung et al., 2009).

Aging population are at a higher risk of suffering from malnutrition due to a general decline in body function including decreased metabolic rate, digestive and absorptive capability (Brownie, 2006). Therefore, the elderly are more likely to be affected by diseases associated with nutritional inadequacy. For example, aging-related vitamin D deficiency has been shown to result in bone loss, susceptibility to fracture, and hyperparathyroidism (Lips, 2001). Therefore, appropriate supplementation with vitamin D can reduce the risk of hip and other fractures in housebound elderly (**Table 1**) (Lips, 2001). In recent years, focus on the diet has increased due to the diet being an essential source of exogenously obtained antioxidants. It appears that dietary antioxidants have the anti-aging activity by their ability to suppress the generation of free radicals (Kandola et al., 2015). Cognitive decline represents a major health concern in aging population (Kang et al., 2005). A key study by Kang et al. (2005) followed over ten thousand women from 1984–2003 to investigate the relationship between their dietary pattern and cognitive function. It was found that women who consumed more green leafy or cruciferous vegetables demonstrated the lowest cognitive decline; while fruit consumption did not affect their cognitive function (Kang et al., 2005). Interestingly, higher intake of green and yellow vegetables was also correlated with a slower rate of skin aging in Japanese women after adjustment for age, BMI, smoking status, and sun exposure (Nagata et al., 2010).

Energy restriction (ER) has recently been put up as a potential way to extend life expectancy. This was partially due to the favorable effects of ER on redox management. In fruit flies, ER diet significantly increased the expression of SOD1 and SOD2 as well as extended lifespan by 16% (Peng et al., 2014). Various natural antioxidants, nutraceuticals, and functional foods have been identified as free radical or progressive oxygen hunters. Therefore, functional foods and nutraceuticals which control the antioxidant activity may represent an important role in slowing the aging process (Peng et al., 2014). A diet rich in antioxidant has been shown to increase lifespan in animal models (Miquel, 2002; Peng et al., 2014). For instance, a diet supplemented blueberry extract was found to markedly improve the lifespan in fruit flies and *Caenorhabditis elegans* (Wilson et al., 2006; Peng et al., 2014). This was accompanied by an increased expression of SOD and catalase. The prolongevity induced by blueberry extract was not observed in SOD or catalase-mutated fruit flies. These results suggest that the beneficial effects of blueberry to extend lifespan are potentially linked with boosted endogenous antioxidant system (Peng et al., 2014). Other nutritional antioxidants including apple polyphenols, black rice anthocyanin extract, and black tea theaflavins all demonstrated prominent prolongevity effects by upregulating the endogenous antioxidant levels in animal models (**Table 1**) (Peng et al., 2014). Further research is needed to evaluate the potential effects of natural antioxidants on life expectancy in human beings.

## Summary and Prospective

The implication of OS in the etiology of several chronic and inflammatory diseases indicates that antioxidant-based therapy could be promising for these disorders. A therapeutic strategy that increases an individual’s antioxidant capacity may be useful for a long-term treatment. However, many problems remain elusive regarding antioxidant supplements in disease prevention. It remains to be elucidated about the precise roles of ROS in the pathogenesis of various diseases. Current recommendations are based on the intake of a healthy diet (rich in fruits, vegetables, and grains and low on red meat and alcohol) and healthy lifestyle, which has demonstrated the ability to reduce the risk for diseases. Further research is warranted before using antioxidant supplements as an adjuvant therapy. In the meantime, avoiding oxidant sources such as cigarette smoke and alcohol must be considered when taking dietary antioxidants.

## Author Contributions

LZ conceptualized and designed the review. ZL, ZR, and JZ summarized the literature and wrote the manuscript. LZ, EK, C-CC, and TZ critically revised the manuscript. TZ prepared the figures and abstract. All authors agreed to be accountable for the content of this work.

## Conflict of Interest Statement

The authors declare that the research was conducted in the absence of any commercial or financial relationships that could be construed as a potential conflict of interest.
